# Investigating antimicrobial prescribing pattern before the development of CDI using data linkage

**DOI:** 10.23889/ijpds.v1i1.312

**Published:** 2017-04-19

**Authors:** Jiafeng Pan, Kim Kavanagh, Charis Marwick, Camilla Wuiff, Peter Davey, Scott Bryson, Chris Robertson, Marion Bennie

**Affiliations:** Department of Mathematics and Statistics, University of Strathclyde; Population Health Sciences, University of Dundee; Health Protection Scotland, NHS National Services Scotland; Strathclyde Institute for Pharmacy and Biomedical Sciences, University of Strathclyde

## Objectives

Prior use of antimicrobials, in particular broad-spectrum antimicrobials, is associated with the development of Clostridium difficile infection (CDI). Our previous work has demonstrated increased risk with cumulative exposure but there is limited evidence on specific patterns of cumulative antimicrobial prescribing prior to infection. Understanding this pattern will help inform antimicrobial stewardship. This study aims to investigate the prescribing patterns for CDI cases with more than 4 weeks cumulative antimicrobial exposure during the 6 months prior to their CDI date.

## Approach

We linked three Scottish patient-level data sets: laboratory confirmed CDI (ECOSS), prescriptions for antimicrobials in primary care (PIS) and all hospital admissions (SMR01). From ECOSS all cases of CDI reported in the period August 2010 to July 2013 were identified. Each CDI case was linked to SMR01 to allocate case type (either hospital or community associated) and to PIS for previous antimicrobial prescriptions. Visual representation of temporal exposure indicating both duration and drug type of each antimicrobial dispensed during the 6 month period before the infection was produced to clearly understand the pattern of prescribing.

## Results

In the study period, there were a total of 1557 community acquired CDI cases without recent (prior 3 months) hospitalisation. Among them, 287 (18%) cases had more than 4 weeks prior cumulative antimicrobial exposure accumulating a total of 1311 dispensed prescriptions. Cases had an average 4 (Q1:3,Q3:6) prescriptions and 2 (Q1:2,Q3:3) different types of antimicrobials dispensed. The timeline plot shows that repeated short duration prescriptions contributed more to cumulative exposure than long duration prophylaxis. The most common antimicrobials prescribed were amoxicillin (38% of cases), trimethoprim (30%) and flucloxacillin (27%). The median duration per prescription for each of these was less than 2 weeks. The antimicrobials that had a median duration of around a month per prescription were oxytetracycline (3%) and clindamycin (6%). 

## Conclusions

This study of a national linked patient level data set contained sufficient numbers of cases to enable investigation of the prescribing pattern in individuals with highest risk cumulative exposure. This study uses NHS Scotland’s developing Infection Intelligence Platform which will place Scotland as a world leader in the use of health informatics to support infection control and antimicrobial stewardship.

